# Criterion of Existence of Power-Law Memory for Economic Processes

**DOI:** 10.3390/e20060414

**Published:** 2018-05-29

**Authors:** Vasily E. Tarasov, Valentina V. Tarasova

**Affiliations:** 1 Skobeltsyn Institute of Nuclear Physics, Lomonosov Moscow State University, 119991 Moscow, Russia; 2 Lomonosov Moscow State University Business School, Lomonosov Moscow State University, 119991 Moscow, Russia

**Keywords:** power-law memory, long memory, economic dynamics, multiplier with memory, accelerator with memory, fractional derivative, fractional integral

## Abstract

In this paper, we propose criteria for the existence of memory of power-law type (PLT) memory in economic processes. We give the criterion of existence of power-law long-range dependence in time by using the analogy with the concept of the long-range alpha-interaction. We also suggest the criterion of existence of PLT memory for frequency domain by using the concept of non-integer dimensions. For an economic process, for which it is known that an endogenous variable depends on an exogenous variable, the proposed criteria make it possible to identify the presence of the PLT memory. The suggested criteria are illustrated in various examples. The use of the proposed criteria allows apply the fractional calculus to construct dynamic models of economic processes. These criteria can be also used to identify the linear integro-differential operators that can be considered as fractional derivatives and integrals of non-integer orders.

## 1. Introduction

In models of economic processes, variables of two types are used. The first type includes exogenous variables that are external to the considered model. The second type includes endogenous variables that are internal to the model. It is possible to state that the separation of variables into exogenous and endogenous ones is based on the causal relationship: the exogenous variable is the cause (the action, input), and the endogenous variable is the consequence (the response, output). In an economic process with memory there is an endogenous variable Y(t) at the time t, which depends on the history of the change of the exogenous variable X(τ) at τ∈(−∞,t). This dependence is due to the fact that people, which participate in the economic process, can remember the previous changes of exogenous variable X(t) and the impact of these changes on the endogenous variable Y(t).

The long-range time dependencies have been empirically observed in economics [1,2,3]. For these dependences the correlations between values of variables decay to zero slower than it can be expected from independent variables or variables from classical Markov and autoregressive moving average models [1,2,3,4,5,6,7]. An interpretation of these dependences between variables is that this process has memory. This property significantly changes the statistical behavior of estimates and predictions in models that take into account memory effects. This leads to the fact, that many of the theoretical models, methods, and approaches, which are used for analyzing classical time series of the Markov and autoregressive moving average processes, cannot be applied to describe processes with memory [1,2,3,4,5,6,7,8,9].

During the development of mathematics, new tools were created to describe processes with memory. There are two main types of methods to analyze the behavior of time series, which describe variables of processes with memory [1,2,3]. The first type is more related to the time-domain analysis such as the correlation analysis. The second type is more connected with an analysis in the frequency domain such as the spectral analysis. Time series, which describe variables of the processes with memory, are characterized by a statistically significant dependence between very distant observations. This dependence is formalized by assuming that the autocorrelation function and spectral densities of these time series decay very slowly, as a function of the time lag. Although there is a general consensus that processes with memory manifest themselves in slowly decaying autocorrelation function and spectral density, there is no universal formal definition of processes with memory [1,2,3]. A stationary process with slowly decaying correlations and spectral density is usually called a process with long memory in contrast to processes with summable correlations, which are also called processes with short memory.

For the first time, the importance of long-range time dependence in economic data was recognized by Clive W. J. Granger in his technical report [10] in 1964, and then in his article [11] in 1966 (see also [12,13,14]). Granger showed that a number of spectral densities, which are estimated from economic time series, have a similar power-law form. Clive W. J. Granger received the Nobel Memorial Prize in Economic Sciences in 2003 “for methods of analyzing economic time series with common trends (cointegration)”. One of the first who used the power law to describe the real economic processes were Charles W. Cobb and Paul H. Douglas, who published the article [15] in 1928. At present time the power laws are widely used in economics, including the Cobb-Douglas functions and other power laws that are described by Xavier Gabaix in the reviews [16,17]. In our paper, we do not consider the criteria for the appearance of power laws in the economy. The main attention is paid only to processes with memory, fading of which has a power-law type. The memory of power-law type will be described in the frequency domain for the exogenous and endogenous variables, between which there is a causal relationship. In this paper, we propose criteria for the existence of memory of power-law type (PLT) memory in economic processes. We suggest criteria for the presence of PLT memory, which make it possible to use the fractional calculus for constructing mathematical models of economic processes with memory. The main attention is paid to processes and models with continuous time, which usually were not considered in standard approaches to economic processes with memory. The standard approach [1,2,3,4,5,6] is based on the fractional differencing and integrating proposed by Granger and Joyeux [14] in 1980, where the discrete time models are used. Note that the fractional differencing and integrating, which are usually used in economics [4,5,6], are the Grunwald–Letnikov fractional differences, which have been proposed more than hundred fifty years ago [8,9]. This fact shows the importance of fractional calculus for modeling processes with memory. It also shows the necessity of criteria for the existence of PLT memory.

## 2. Processes with Memory

We assume that an economic process with memory is characterized by the fact that there is an endogenous variable Y(t) at the time t, which depends on the history of the change of the exogenous variable X(τ) at τ∈(−∞,t) [18,19,20]. In this case, interrelations of these endogenous and exogenous variables can be described by concepts of economic multiplier with memory or/and accelerator with memory, which have been proposed in [21,22,23,24] and applied to different economic models [25,26,27,28,29,30].

Let us assume that the real functions Y(τ) and X(τ) are given and these functions describe the endogenous variable and the exogenous variable for τ∈(−∞,t] of an economic process, respectively. We also assume that it is known existence of dependence an endogenous variable on the exogenous variable. We are interested in the possibility of determining the presence of memory in this process. To formulate criteria of existence of memory in the frequency domain, we consider the Fourier transform F of the given functions Y(τ) and X(τ), which we denote by $\tilde{Y}\left(\omega \right)=F\left\{Y\left(t\right)\right\}\left(\omega \right),$ and $\tilde{X}\left(\omega \right)=F\left\{X\left(t\right)\right\}\left(\omega \right).$ The Fourier transform will be defined by the equation:

(1) $$\tilde{X}\left(\omega \right)=F\left\{X\left(t\right)\right\}\left(\omega \right)=\int_{-\infty }^{+\infty }X\left(t\right)\cdot e^{i\cdot \omega \cdot t}\mathrm{dt},$$

where we use a positive sign in front i·ω·t as in [31] (p. 24) and [32] (p. 10), [33] (pp. 109–112). The inverse Fourier transform $F^{-1}$ is given by the formula

(2) $$X\left(t\right)=F^{-1}\left\{\tilde{X}\left(\omega \right)\right\}\left(t\right)=\frac{1}{2\pi }\int_{-\infty }^{+\infty }\tilde{X}\left(\omega \right)\cdot e^{-i\cdot \omega \cdot t}d\omega .$$

The integrals in Equations (1) and (2) converge absolutely for functions $X\left(t\right),\tilde{X}\left(\omega \right)\in L_{1}\left(R\right)$ and in the norm of the space $L_{2}\left(R\right)$ for $X\left(t\right),\tilde{X}\left(\omega \right)\in L_{2}\left(R\right)$.

In the frequency domain, we can define the function

(3) $$\tilde{M}\left(\omega \right)=\frac{\tilde{Y}\left(\omega \right)}{\tilde{X}\left(\omega \right)}$$

for frequencies ω∈ℝ at which the function $\tilde{X}\left(\omega \right)$ is nonzero, $\tilde{X}\left(\omega \right)\neq 0$. The function $\tilde{M}\left(\omega \right)$ will be called the frequency memory function or the frequency response function. Fundamentally, the frequency memory function is a mathematical representation of the relationship between the exogenous and the endogenous variables. Here we consider the frequency memory function for single exogenous variable  X(t) and single endogenous variable Y(t).

Since the functions Y(τ) and X(τ) are real, we have the equalities ${\tilde{X}}^{*}\left(\omega \right)=\tilde{X}\left(-\omega \right)$ and ${\tilde{Y}}^{*}\left(\omega \right)=\tilde{Y}\left(-\omega \right)$. This leads to the property ${\tilde{M}}^{*}\left(\omega \right)=\tilde{M}\left(-\omega \right)$.

In the general case, the frequency dependence of $\tilde{M}\left(\omega \right)$ defines the memory function $M\left(t\right)=F^{-1}\left\{\tilde{M}\left(\omega \right)\right\}\left(t\right)$ that describes a non-local connection between the endogenous variable Y(τ) and the exogenous variable X(τ) in time domain.

We assume that the memory function does not depend on the explicit form of the functions of the exogenous and endogenous variables. Symbolically, we will write this in the form $\tilde{M}\left(\omega ,\tilde{X}\left(\omega \right),\tilde{Y}\left(\omega \right)\right)=\tilde{M}\left(\omega \right)$. The memory function depends on the type of considered economic process. In other words, we will assume that the memory function characterizes the memory effects, and it is independent of the behavior of the exogenous or endogenous variable. The memory function only reveals properties of economic processes if it is known that the variable Y depends linearly on the variable X. We also assume that the economic process under consideration is characterized by the dependence of an endogenous variable Y on the change of the exogenous variable X. Using this assumption, we propose criteria can be used to identify the memory of power-law type in economic processes. These criteria allow us to say for which processes we should use more general economic models base on fundamental concepts of multiplier and accelerator with memory [21,22,23,24].

Before we proceed to the formulation of the criterion for the existence of memory in the economic process, we will explain the main aspects of the proposed approach by the simplest examples.

It is well-known that fractional derivatives and integrals of non-integer order can describe a memory. Fractional derivatives and integrals of integer orders cannot describe a memory since equations with these operators can be represented by differential equations of integer orders [34]. As an example, let us consider the fractional integral equation

(4) $$Y\left(t\right)=m_{1}\cdot (I_{L;+}^{\alpha_{1}}X)\left(t\right)+m_{2}\cdot (I_{L;+}^{\alpha_{2}}X)\left(t\right),$$

where $\alpha_{2}>\alpha_{1}>0$ and $I_{L;+}^{\alpha }$ is the left-sided Liouville integral of the order α > 0 with respect to time variable, $m_{1}$ and $m_{2}$ are numerical coefficients. In economics, Equation (4) describes a linear multiplier with memory [18,19,21,22,23,24]. Note that the terms with integrals of integer order do not describe a memory, since by differentiating of this order we obtain a differential equation of integer order. For example, for $\alpha_{2}=1$ and $\alpha_{1}$ is non-integer, differentiation of Equation (4) gives:

(5) $$Y\left(t\right)=m_{1}\cdot (D_{L;+}^{1-\alpha_{1}}X)\left(t\right)+m_{2}\cdot X\left(t\right).$$

Therefore memory is described by first term of order $\alpha_{1}$. The following question arises: How we can identify this memory that is described by non-integer $\alpha_{1}$, when $\alpha_{2}$ is integer ($\alpha_{2}=1$)? Using the Fourier transform of the left-sided Liouville integral [32] (p. 90), we get

(6) $$\tilde{Y}\left(\omega \right)=\tilde{M}\left(\omega \right)\cdot \tilde{X}\left(\omega \right),$$

where

(7) $$\tilde{M}\left(\omega \right)=m_{1}\cdot {\left(-i\omega \right)}^{-\alpha_{1}}+m_{2}\cdot {\left(-i\omega \right)}^{-\alpha_{2}},$$

(8) $${\left(-i\omega \right)}^{\alpha }={\left|\omega \right|}^{\alpha }\cdot \exp\left(-i\cdot \alpha \cdot \pi \cdot \frac{\mathrm{sgn}\left(\omega \right)}{2}\right).$$

This representation allows us to get the limits

(9) $$\alpha_{0}:=\underset{\omega \to 0}{\lim}\frac{\ln\left|\tilde{M}\left(\omega \right)\right|}{\ln\left|\omega \right|}=-\alpha_{2},$$

(10) $$\alpha_{\infty }:=\underset{\omega \to \infty }{\lim}\frac{\ln\left|\tilde{M}\left(\omega \right)\right|}{\ln\left|\omega \right|}=-\alpha_{1},$$

i.e., $\alpha_{0}=-\alpha_{2}=-1$ and $\alpha_{\infty }=-\alpha_{1}$ is non-integer. As a result, we see that the limit with ω goes to zero (ω→0) cannot identify this memory. We also can see that the limit with ω goes to infinity (ω→∞) can be realized this identification. The limits (9) and (10), which allow us to identify the PLT memory, can be used to formulate a criterion of existence of PLT memory for frequency domain. Using the function $\tilde{M}\left(\omega \right)$, the existence of a memory can be connected with the existence of the limits (9) and (10), i.e., with non-integer values of the parameters $\alpha_{0}$ and/or $\alpha_{\infty }$.

Due to the symmetry of the behavior of the Liouville fractional integral, the memory (7) with $\alpha_{2}=1$ and $0<\alpha_{1}<1$ can also be identified by another way as described in Section 4 by using the Criterion of existence of the next-to-order values of memory and considering ω→0. Let us consider the limit

(11) $$A_{0}\left(\alpha_{0}\right):=\underset{\omega \to 0+}{\lim}\frac{\tilde{M}\left(\omega \right)}{\omega^{\alpha_{0}}}.$$

For the Function (7), Equation (11) with $\alpha_{0}=-\alpha_{2}=-1$ gives $A_{0}\left(\alpha_{0}\right)=m_{2}\cdot {\left(-i\right)}^{-\alpha_{2}}=i\cdot m_{2}$. Then we consider the next-to-order values of the limits:

(12) $$\alpha_{0,1}:=\underset{\omega \to 0+}{\lim}\frac{\ln\left|\tilde{M}\left(\omega \right)-A_{0}\left(\alpha_{0}\right)\cdot \omega^{\alpha_{0}}\right|}{\ln\left|\omega \right|}=-\alpha_{1},$$

The limits (9) and (10), which allow us to identify the PLT memory, can be used to formulate a criterion of the next-to-order values of PLT memory fading parameters for frequency domain.

Note that in the considered Example (4), we can identify the memory by the next-to-order limit (12) since the Fourier transform of the Liouville fractional integral has the symmetry of the behavior (the same behavior when ω→0 and ω→∞), i.e., for Equations (4) and (7) with $m_{2}=0$, we have:

(13) $$\tilde{M}\left(\omega \right)\sim {\left(-i\omega \right)}^{-\alpha_{1}},\mathrm{for}\;\omega \to \infty ,$$

(14) $$\tilde{M}\left(\omega \right)\sim {\left(-i\omega \right)}^{-\alpha_{1}}\;\mathrm{for}\;\omega \to 0\;.$$

However, when formulating the criterion for the existence of memory, we must also take into account asymmetric cases. For example, In the Jonscher universal response phenomenon for the branches of dielectric spectra [35,36,37,38] (see also [39,40,41,42,43,44,45,46]), the power-law behavior for ω→∞ should be considered in addition to ω→0 to identify a memory because of asymmetric behavior.

The power-law type of behavior of function $\tilde{M}\left(\omega \right),$ which describes complex susceptibility of dielectric media, has been used by Jonscher [35,36,37,38] to formulate the “universal” response phenomenon, when the behavior of dielectric spectra are described by the expressions:

(15) $$\tilde{M}\left(\omega \right)-\tilde{M}\left(0\right)\sim -{\left(i\cdot \omega \right)}^{\beta },\mathrm{for}\;\omega \to 0\;\left(\omega \ll \omega_{p}\right),$$

(16) $$\tilde{M}\left(\omega \right)\sim {\left(i\cdot \omega \right)}^{-\alpha }\;\mathrm{for}\;\omega \to \infty \;\left(\omega \gg \omega_{p}\right),$$

where α and β fall in the range (0;1). The “universal” response is based on the experimental observation of power laws at frequencies far from the peak frequency $\omega_{p}$ of the imaginary part ${\tilde{M}}_{2}\left(\omega \right)$ of the function $\tilde{M}\left(\omega \right)$. The asymmetric behavior of the form (15) and (16) can be described [35] by the function $\tilde{M}\left(\omega \right)$ with the imaginary part:

(17) $${\tilde{M}}_{2}\left(\omega \right)=\frac{1}{{\left(\omega /\omega_{p}\right)}^{\alpha }+{\left(\omega /\omega_{p}\right)}^{-\beta }}$$

where $\omega_{p}$ is the loss peak frequency. The real part of $\tilde{M}\left(\omega \right)$ is expresses through the imaginary part (14) by the Kramers–Kronig relation [47,48,49,50,51,52,53,54,55]. The behavior of this memory function $\tilde{M}\left(\omega \right)$ is described by (15) and (16). As a result, in the case β=1 and non-integer value α>0 of the PLT memory cannot be identified by the limit ω→0. For non-integer values of α and integer β>0 the behavior at ω→∞ cannot be interpreted as a long memory, but it should be interpreted as a memory. This memory will be called the power-law type memory (the PLT memory). This concept includes the long memory as a special case.

In addition to application of the proposed criteria to describe processes with memory in natural sciences, economic and finance, these criteria can be applied for pure mathematical purpose of development of fractional calculus. It is known that the power-law memory function allows us the use of fractional calculus to describe processes with memory. In recent years some different type of new integral and differential operators are proposed. Some of these operators cannot be used to describe a memory since it cannot be considered as non-local operators [34]. Note that memory can be interpreted as a nonlocality is time. Therefore we can state “No memory. No fractional derivatives and integrals” by analogy with [34]. In this connection, the question arises whether it is possible to consider a wider class of functions than power-law function (we will call these functions as power-law type), which can be used to describe a memory, and allows us to use the fractional calculus or its generalization. The proposed criteria can be used to identify the linear integro-differential operators that can be considered as fractional derivatives and integrals of non-integer order. It allows to propose new type of fractional derivatives and integrals, and as a result this will allow expanding the area of generalized fractional calculus [56,57].

## 3. Criteria of Existence of Power-Law Type Memory in Economic Process

In this section, we give the criterion of existence of a long-range dependence in time by using the concept of the long-range alpha-interaction and the criterion of existence of PLT memory for frequency domain by using the concept of non-integer dimensions.

In [58,59] and [60] (p. 166) the concept of the long-range alpha-interaction has been proposed. This concept describes power-law non-locality in space. The concept of memory can be interpreted as a non-locality in time. It allows us to use the concept of the long-range alpha-interaction [58,59,60] (see also [61,62]) in order to formulate the following criterion of the memory of power-law type (the PLT memory). The difference between these types of nonlocality is that we must take into account the causality for the concept of nonlocality in time. The causality will be discussed in Section 5 of this paper.

Let us define the function:

(18) $${\tilde{M}}_{0}\left(\omega \right)=\{\begin{array}{c} \tilde{M}\left(\omega \right)-\tilde{M}\left(0+\right),\\ \tilde{M}\left(\omega \right) \end{array}\begin{array}{c} \mathrm{if}\;0<\left|\tilde{M}\left(0+\right)\right|<\infty ,\\ \mathrm{in}\;\mathrm{other}\;\mathrm{cases}, \end{array}$$

where $\tilde{M}\left(\omega \right)$ is defined by Equation (3) and

(19) $$\tilde{M}\left(0+\right)=\underset{\omega \to 0+}{\lim}\tilde{M}\left(\omega \right).$$

Using the memory function in the frequency domain (3) and the concept of the long-range alpha-interaction, we can formulate the criterion of existence of long-range dependence in time. For an economic process, for which it is known that an endogenous variable Y(t) depends on the exogenous variable X(t), we can formulate the following criterion of the existence of the PLT memory. 

### 3.1. Criterion of Existence of Long-Range Dependence in Time

Let for the considered economic process it is known that the endogenous variable Y(t) depends on an exogenous variable X(t). This economic process is characterized by memory of the power-law type if there exists a non-integer parameter $\alpha_{0}$ and there exists the finite limit

(20) $$\underset{\omega \to 0+}{\lim}\frac{{\tilde{M}}_{0}\left(\omega \right)}{\omega^{\alpha_{0}}}=A_{0}\left(\alpha_{0}\right),$$

where $0<\left|A_{0}\left(\alpha_{0}\right)\right|<\infty$ and ${\tilde{M}}_{0}\left(\omega \right)$ is defined by Equation (18).

Similarly, we can consider the existence of a non-integer parameter $\alpha_{\infty }$ and the finite limit at ω→∞ in the form

(21) $$\underset{\omega \to \infty }{\lim}\frac{{\tilde{M}}_{\infty }\left(\omega \right)}{\omega^{\alpha_{\infty }}}=A_{\infty }\left(\alpha_{\infty }\right),$$

where $0\langle |A_{\infty }\left(\alpha_{\infty }\right)|<\infty$ and the function ${\tilde{M}}_{\infty }\left(\omega \right)$ is defined by the equation

(22) $${\tilde{M}}_{\infty }\left(\omega \right)=\{\begin{array}{c} \tilde{M}\left(\omega \right)-{\tilde{M}}_{\infty }\\ \tilde{M}\left(\omega \right) \end{array}\begin{array}{c} \mathrm{if}\;0<\left|{\tilde{M}}_{\infty }\right|<\infty ,\\ \mathrm{in}\;\mathrm{other}\;\mathrm{cases}, \end{array}$$

and ${\tilde{M}}_{\infty }=\underset{\omega \to \infty }{\lim}\tilde{M}\left(\omega \right).$

For economic processes, which are characterized by dependence of an endogenous variable Y(t) on the exogenous variable X(t), we can formulate new criteria that allow us to take into account the multi-parametric memory of power-law type. We propose a criterion of the presence of PLT memory, which describes a certain type of nonlocality in time, by using some analogy with non-integer physical dimension of space [60].

Using the Function (3), the existence of a memory can be connected with the existence of the finite limits for (18) and (22), which are equal to parameters $\alpha_{0}$ and/or $\alpha_{\infty }$.

### 3.2. Criterion of Existence of PLT Memory (for Frequency Domain)

Let for the considered economic process it is known that the endogenous variable Y(t) depends on an exogenous variable X(t). In this economic process there is a memory of power-law type if at least one of the limits

(23) $$\underset{\omega \to 0}{\lim}\frac{\ln\left|{\tilde{M}}_{0}\left(\omega \right)\right|}{\ln\left|\omega \right|}=\alpha_{0},$$

(24) $$\underset{\omega \to \infty }{\lim}\frac{\ln\left|{\tilde{M}}_{\infty }\left(\omega \right)\right|}{\ln\left|\omega \right|}=\alpha_{\infty }$$

takes a non-integer value, where ${\tilde{M}}_{0}\left(\omega \right)$ and ${\tilde{M}}_{\infty }\left(\omega \right)$ are defined by Equations (18) and (22), respectively.

The suggested criterion associates the presence of memory with parameters of nonlocality in time, which can be interpreted as the fading parameters of PLT memory [18,19].

There is another interpretation of the parameters $\alpha_{0}$ and $\alpha_{\infty }$. These parameters can be interpreted as non-integer dimensions of time by analogy with the non-integer dimension of space (for example, see [63,64,65] and [66] (pp. 62–87)). This “time dimensions” is a characteristic of set of histories. This interpretation is based on the similarity of Equations (23), (24) and equations, which determine the physical dimensions of the mass, charge and number of particles [60].

We note that any of the inequalities $\alpha_{\infty }\geq \alpha_{0}$ or $\alpha_{\infty }\leq \alpha$ can be realized. The parameter $\alpha_{\infty }$ does not need to be greater than the parameter $\alpha_{0}$, which can erroneously seem from the notation.

For the existence of PLT memory, it is necessary that at least one of the parameters has a non-integer value. If there are both parameters $\alpha_{0}$ and $\alpha_{\infty }$ are non-integer and $\alpha_{\infty }\neq \alpha_{0}$, then we have the multi-parametric memory of power-law type.

Note that the integer values of the parameters (23) and (24) do not mean that there is no memory of the power type in the economic process. For such conclusions, it is necessary to make sure that there are no other parameters. To find new parameters we should consider new limits, when the power-law dependences with $\alpha_{0}$ and $\alpha_{\infty }$ are subtracted. For this purpose, we consider generalizations of the suggested criteria. In the next section, proposed criteria will be generalized to take into account the other parameters for the multi-parametric memory of power-law type.

## 4. Criteria of Multi-Parametric Memory of Power-Law Type

First, we consider a criterion for the existence of the next-to-order values of memory fading parameters. It allows us to formulate the criterion that takes into account all possible parameters for the multi-parametric memory of power-law type.

### 4.1. Criterion of Existence of the Next-to-Order Values of Memory Fading Parameters

In the economic process, for with it is known that an endogenous variable Y(t) depends on exogenous variables X(t), there is a multi-parametric memory of power-law type if there are at least two of the limits (23)–(24) and

(25) $$\underset{\omega \to 0+}{\lim}\frac{\ln\left|{\tilde{M}}_{0}\left(\omega \right)-A_{0}\left(\alpha_{0}\right)\cdot \omega^{\alpha_{0}}\right|}{\ln\left|\omega \right|}=\alpha_{0,1},$$

(26) $$\underset{\omega \to \infty }{\lim}\frac{\ln\left|{\tilde{M}}_{\infty }\left(\omega \right)-A_{\infty }\left(\alpha_{\infty }\right)\cdot \omega^{\alpha_{\infty }}\right|}{\ln\left|\omega \right|}=\alpha_{\infty ,1}$$

take a non-integer value, where $A_{0}\left(\alpha_{0}\right)$ and $A_{\infty }\left(\alpha_{\infty }\right)$ are defined by Equations (20) and (21).

Let us note that parameters $\alpha_{0,1}$ and $\alpha_{\infty ,1}$ define the following in the seniority of the smallest $\alpha_{0}$ and the largest $\alpha_{\infty }$, respectively. If all four parameters exist and $\alpha_{\infty }>\alpha_{0}$, then inequality $\alpha_{\infty }>\alpha_{\infty ,1}\geq \alpha_{0,1}>\alpha_{0}$ holds. Similarly, it is possible to determine all subsequent parameters of extinction of multi-parametric memory of power-law type.

Then, we can consider the limits

(27) $$\underset{\omega \to 0+}{\lim}\frac{{\tilde{M}}_{0}\left(\omega \right)-A_{0}\left(\alpha_{0}\right)\cdot \omega^{\alpha_{0}}}{\omega^{\alpha_{0,1}}}=A_{0,1}\left(\alpha_{0,1}\right),$$

(28) $$\underset{\omega \to 0+}{\lim}\frac{{\tilde{M}}_{\infty }\left(\omega \right)-A_{\infty }\left(\alpha_{\infty }\right)\cdot \omega^{\alpha_{\infty }}}{\omega^{\alpha_{\infty ,1}}}=A_{\infty ,1}\left(\alpha_{\infty ,1}\right).$$

Using these limits, we can define the parameters

(29) $$\underset{\omega \to 0+}{\lim}\frac{\ln\left|{\tilde{M}}_{0}\left(\omega \right)-A_{0}\left(\alpha_{0}\right)\cdot \omega^{\alpha_{0}}-A_{0,1}\left(\alpha_{0,1}\right)\cdot \omega^{\alpha_{0,1}}\right|}{\ln\left|\omega \right|}=\alpha_{0,2},$$

(30) $$\underset{\omega \to \infty }{\lim}\frac{\ln\left|{\tilde{M}}_{\infty }\left(\omega \right)-A_{\infty }\left(\alpha_{\infty }\right)\cdot \omega^{\alpha_{\infty }}-A_{\infty ,1}\left(\alpha_{\infty ,1}\right)\cdot \omega^{\alpha_{\infty ,1}}\right|}{\ln\left|\omega \right|}=\alpha_{\infty ,2}.$$

Continuing by analogy, one can find all the memory parameters of the power type. It allows us to formulate the following criterion of multi-parametric memory.

### 4.2. Criterion of Existence of Multi-Parametric Memory (for Frequency Domain)

In the economic process, for with it is known that an endogenous variable Y(t) depends on exogenous variables X(t), there is a multi-parametric PLT memory if there are at least two of the limits

(31) $$\underset{\omega \to 0+}{\lim}\frac{\ln\left|{\tilde{M}}_{0}\left(\omega \right)-\sum_{k=0}^{m}A_{0,k}\left(\alpha_{0,k}\right)\cdot \omega^{\alpha_{0,k}}\right|}{\ln\left|\omega \right|}=\alpha_{0,m+1},$$

(32) $$\underset{\omega \to \infty }{\lim}\frac{\ln\left|{\tilde{M}}_{\infty }\left(\omega \right)-\sum_{k=0}^{n}A_{\infty ,k}\left(\alpha_{\infty ,k}\right)\cdot \omega^{\alpha_{\infty ,k}}\right|}{\ln\left|\omega \right|}=\alpha_{\infty ,n+1},$$

where $A_{0,k}\left(\alpha_{0,k}\right)$ and $A_{\infty ,k}\left(\alpha_{\infty ,k}\right)$ are defined by the equations

(33) $$A_{0,s}\left(\alpha_{0,q+1}\right)=\underset{\omega \to 0+}{\lim}\frac{{\tilde{M}}_{0}\left(\omega \right)-\sum_{k=0}^{q}A_{0,k}\left(\alpha_{0,k}\right)\cdot \omega^{\alpha_{0,k}}}{\omega^{\alpha_{0,q}}},$$

(34) $$A_{\infty ,s}\left(\alpha_{\infty ,p+1}\right)=\underset{\omega \to 0+}{\lim}\frac{{\tilde{M}}_{\infty }\left(\omega \right)-\sum_{k=0}^{p}A_{\infty ,k}\left(\alpha_{\infty ,k}\right)\cdot \omega^{\alpha_{\infty ,k}}}{\omega^{\alpha_{\infty ,p+1}}},$$

and we use the notations $A_{0,0}\left(\alpha_{0,0}\right)=A_{0}\left(\alpha_{0}\right),$ $A_{\infty ,0}\left(\alpha_{\infty ,0}\right)=A_{\infty }\left(\alpha_{\infty }\right)$ and $\alpha_{0,0}=\alpha_{0}$, $\alpha_{\infty ,0}=\alpha_{\infty }$. Numbers m and n run through all possible non-negative values, for which the parameters $\alpha_{0,m+1}$ and $\alpha_{\infty ,n+1}$ are non-zero.

Let us suppose that there are only parameters $\alpha_{\infty }$, $\alpha_{0}$ and there are no other parameters ($\alpha_{0,k}$, $\alpha_{\infty ,k}$), k≥1, k∈ℤ. In this case, the existence of parameters $\alpha_{\infty }$, $\alpha_{0}$ can be interpreted as follows.

The existence of these parameters means the existence of equation, which describes the economic accelerators and/or multipliers that relates the exogenous and endogenous variables. For non-integer positive values of parameters $\alpha_{0}>0$ and $\alpha_{\infty }>0$, we have the economic accelerator with multi-parametric power-law memory. If parameters are equal to each other $\alpha_{0}=\alpha_{\infty }>0$ and this value is positive non-integer, then we have an accelerator with one-parametric PLT memory.

For non-integer negative values of parameters ($\alpha_{0}<0$ and $\alpha_{\infty }<0$), we have the economic multiplier with multi-parametric power-law memory. If parameters are equal to each other $\alpha_{0}=\alpha_{\infty }<0$ and this value is positive non-integer, then we have the multiplier with one-parametric PLT memory [18,19,20,21].

If these parameters have non-integer values and different signs ($\alpha_{\infty }>0>\alpha_{0}$ or $\alpha_{0}>0>\alpha_{\infty }$), then we have an accelerator-multiplier with PLT memory, that is, a combined effect of an accelerator and a multiplier with PLT memory. To illustrate the described properties, we give the following Table 1.

If both parameters are equal to one ($\alpha_{0}=\alpha_{\infty }=1$) and there are no other non-integer parameters, then we have the phenomenon of the accelerator without PLT memory. If both parameters are equal to zero ($\alpha_{0}=\alpha_{\infty }=0$) and there are no other non-integer parameters, then we have the phenomenon of multiplier without PLT memory.

## 5. Causality Principle for Economic Processes with Memory

The separation of variables into exogenous and endogenous ones is based on the causal relationship: the exogenous variable is the cause (the action), and the endogenous variable is the consequence (the response). The exogenous and endogenous variables can also be described as input and output. In the general case, the causal relationship assumes a finite rate of process and implies the influence of some economic processes on others.

In general, we should be very careful to delimit the concept of causality, in order to distinguish it from controllability and exogeneity [67,68,69,70,71,72,73,74,75,76,77].

The history of the concept of causality in economics is described by Hoover in the article “Causality in economics and econometrics” [67], (see also [68,69]).

An important concept of causality was proposed by Granger in 1969 [70], (see also [13,71]). The main assumption of Granger was that the future cannot be the cause of the present or the past. This means that the cause must at least precede the effect. However, the very fact of precedence does not say anything about the existence of a causal relationship. The importance of the fact that the preceding values of the “cause” should have a perceptible influence on the future values of the “effect” and, moreover, the past values of the “effect” did not have a significant effect on the future values of the “cause”. The concept of the Granger causality is used in econometrics and time series analysis to formalize the notion of a cause-effect relationship between time series [13,70,71]. The Granger causality is necessary, but not sufficient, for a cause-effect relationship.

Clive Granger [71] (p. 330) proposes the following definition of causality that takes into account the memory effect in the form of the past histories: a variable X(t) causes Y(t), if the probability of Y(t) conditional on its own past history and the past history of X(t) (besides the set of the available information) does not equal the probability of Y(t) conditional on its own past history alone. In the economic literature this definition is called the Granger-causality, which is applied in econometrics. The Granger-causality is formulated for the time domain.

The concept of causality often appears in discussion of the interpretation of a correlation coefficient or regression, since an observed relationship does not allow us to say anything about causation between the variables.

Unfortunately, is no generally accepted procedure for testing for causality, partially, because of a lack of the definition of this concept that can be universal and can be accepted by all.

In this section we describe the causality for economic processes with memory in the time and frequency domains.

### 5.1. The Causality Principle for Memory: Time Domain

The function $\tilde{M}\left(\omega \right)$ can be considered as the Fourier transform $\tilde{M}\left(\omega \right):=F\left\{M\left(t\right)\right\}\left(\omega \right)$ of a function M(t). The function $M\left(t\right)≔F^{-1}\left\{\tilde{M}\left(\omega \right)\right\}\left(t\right)$ can be interpreted as a response function. In linear case, the response function describes how some time-dependent property Y(t) of an economic process responds to an impulse impact X(t).

The principle of “strict causality” implies the following statement “no output can occur before the input” [72]. Therefore, the response M(t−τ) must be zero for t< τ since a process cannot react to the impact before it is applied. It can be shown (for instance, by invoking Titchmarsh’s theorem [78]) that this causality condition implies that the Fourier transform $\tilde{M}\left(\omega \right)$ of M(t) is analytic in the upper half plane.

Let us consider the dependence of Y(t) from X(τ) that takes into account the memory in the time domain. For example, this dependence, which takes into account the “strict causality”, can be described [15,16,18,19] by the integral equation

(35) $$Y\left(t\right)=\int_{-\infty }^{t}M\left(t-\tau \right)\cdot X\left(\tau \right)d\tau ,$$

or the integro-differential equation

(36) $$Y\left(t\right)=\frac{d^{n}}{{\mathrm{dt}}^{n}}\int_{-\infty }^{t}M\left(t-\tau \right)\cdot X\left(\tau \right)d\tau ,$$

where M(t−τ) is the memory function that allows you to take into account the memory in economic processes.

Suppose that an endogenous variable Y(t) at the time t depends on another (for example exogenous) variable X(τ) at all other times τ by the linear relationship

(37) $$Y\left(t\right)=\int_{-\infty }^{+\infty }R\left(t-\tau \right)\cdot X\left(\tau \right)d\tau ,$$

where R(t−τ) is a response function. For R(t−τ)=m·δ(t−τ), Equation (37) gives Y(t)=m·X(t) that is the standard multiplier equation, where m is the multiplier coefficient.

From the convolution theorem, the Fourier transforms from time t to frequency ω of Equation (37) gives the following relationship for frequency domain in the form

(38) $$\tilde{Y}\left(\omega \right)=\tilde{R}\left(\omega \right)\cdot \tilde{X}\left(\omega \right),$$

where $\tilde{Y}\left(\omega \right)=F\left\{Y\left(t\right)\right\}\left(\omega \right),\tilde{X}\left(\omega \right)=F\left\{X\left(t\right)\right\}\left(\omega \right),$ and $\tilde{R}\left(\omega \right)=F\left\{R\left(t\right)\right\}\left(\omega \right).$ Comparing Expression (38) with the definition of the function $\tilde{M}\left(\omega \right)$ in Equation (3), we can see that the response function can be expressed through the memory function. 

Strict causality, which means that the past can determine the present, but the future cannot, is expressed as R(t−τ)=0 for τ>t. This allows us to represent the response function in the form

(39) R(t−τ)=H(t−τ)·M(t−τ),

where H(t−τ) is the Heaviside step function that is defined as

(40) $$H\left(t-\tau \right)=\{\begin{array}{c} 1,\\ 0, \end{array}\;\begin{array}{c} t-\tau >0;\\ t-\tau <0. \end{array}$$

The appearance of the Heaviside step function in the definition of the response function reminds us that the endogenous variable Y(t) can only depend on the past values of the exogenous variable X(τ), where X(τ) can be considered as an external impact on the process and Y(t) can be considered as the response of the process to this impact. Therefore, (37) gives a causal relationship between the endogenous variable Y(t) and the exogenous variable X(τ) in the form

(41) $$Y\left(t\right)=\int_{-\infty }^{t}M\left(t-\tau \right)\cdot X\left(\tau \right)d\tau .$$

This equation can be considered as a generalized linear multiplier with memory.

Note that the response R(t−τ) must be zero for t< τ since a process cannot react to the impact before it is applied. It can be shown (for instance, by invoking Titchmarsh’s theorem) that this causality condition in the frequency domain implies that the Fourier transform $\tilde{M}\left(\omega \right)$ of M(t) is analytic in the upper half plane. 

### 5.2. The Causality Principle for Memory: Frequency Domain

To describe causality in the economics with memory, we will use the econophysics approach. For economic processes with memory, the causality can be described by the Kramers–Kronig relations [47,48,49,50,51,52,53,54,55]. Under suitable mathematical conditions, the principle of causality in the time domain can be represented in the form of the Kramers–Kronig relations (the Hilbert transform pair) by the Fourier transforms. The Kramers–Kronig relations represent the principle of causality in the frequency domain. The Kramers–Kronig relations mean that the real part M˜1(ω)=Re[M˜(ω)] and the imaginary parts M˜2(ω)=Im[M˜(ω)] of the memory function are not independent, and the full function can be reconstructed given just one of its parts. In general, $\tilde{M}\left(\omega \right)$ is the complex function $\tilde{M}\left(\omega \right)={\tilde{M}}_{1}\left(\omega \right)+i\cdot {\tilde{M}}_{2}\left(\omega \right),$ where function, where M˜1(ω)=Re[M˜(ω)] and M˜2(ω)=Im[M˜(ω)] are real-valued functions.

Suppose the function $\tilde{M}\left(\omega \right)$ is analytic in the closed upper half-plane of frequency ω and vanishes like 1/|ω| or faster as |ω|→∞. Slightly weaker conditions are also possible. The Kramers–Kronig relations are given by:

(42) $${\tilde{M}}_{1}\left(\omega \right)=\frac{1}{\pi }P.V.\overset{+\infty }{\underset{-\infty }{\int }}\frac{1}{\Omega -\omega }{\tilde{M}}_{2}\left(\Omega \right)d\Omega ,$$

(43) $${\tilde{M}}_{2}\left(\omega \right)=-\frac{1}{\pi }P.V.\overset{+\infty }{\underset{-\infty }{\int }}\frac{1}{\Omega -\omega }{\tilde{M}}_{1}\left(\Omega \right)d\Omega ,$$

where P.V. denotes the Cauchy principal value. These relations are also called the dispersion relations. They were first derived by Kramers (1927) and de L. Kronig (1926) in physics independently [47,48,49]. The real part of a response function describes how a system dissipates “energy”, since it is in phase with the driving force. The Kramers–Kronig relations imply that the observation of the dissipative response of a system is sufficient to determine its out-of-phase (reactive) response and vice versa.

In the Kramers–Kronig relations, the integrals run from −∞ to +∞, implying we know the response at negative frequencies. In many casses, the positive frequency-response determines the negative-frequency response because $\tilde{M}\left(\omega \right)$ is the Fourier transform of a real-valued function quantity M(t). As a consequence, we have $\tilde{M}\left(-\omega \right)={\tilde{M}}^{*}\left(\omega \right)$. This means ${\tilde{M}}_{1}\left(\omega \right)$ is an even function of frequency (${\tilde{M}}_{1}\left(-\omega \right)={\tilde{M}}_{1}\left(\omega \right)$) and ${\tilde{M}}_{2}\left(\omega \right)$ is odd (${\tilde{M}}_{2}\left(-\omega \right)=-{\tilde{M}}_{2}\left(\omega \right)$).

The Kramers–Kronig relations can be derived by two approaches, which use the Fourier representation of the Heaviside step function and the freedom to define the memory function M(t−τ) for t−τ<0, respectively [50]. More traditional derivation of the Kramers–Kronig relations is usually suggested for electrodynamics of continuous media (for example, see Section 7.10 of [51] (pp. 330–335)). The traditional method of proving these relations is to continue $\tilde{M}\left(\omega \right)\;$ to complex frequencies and then to exploit its analyticity in the upper half of the ω plane. Historical description of how the Kramers–Kronig relations were first derived is proposed in [49].

In general, it is difficult to prove the equivalence between the Kramers–Kronig relations (KKR) and the causality in time domain (CTD). If $\tilde{M}\left(\omega \right)$ is a square-integrable function, then the Titchmarsh theorem [78] guarantees that KKR and CTD are equivalent. The square-integrability requirement on $\tilde{M}\left(\omega \right)$ can be weakened. It can be shown that if $\tilde{M}\left(\omega \right)$ is bounded but Y(t) and X(τ) are square-integrable functions, then KKR and CTD are equivalent [72]. Unfortunately, these conditions are generally not satisfied. Therefore, we should consider R(t) and M(t) as generalized functions (distributions) and not as classical functions.

The derivation of the Kramers–Kronig relations can be based on two well-known results [50]. The first is the Fourier transform of a convolution. The second is the fact that the Fourier transform of the step function H(t) has the form

(44) $$\tilde{H}\left(\omega \right)=F\left\{H\left(t\right)\right\}\left(\omega \right)=\int_{-\infty }^{+\infty }H\left(t\right)\cdot e^{-i\cdot \omega \cdot t}\mathrm{dt}=\underset{\varepsilon \to 0+}{\lim}\frac{i}{\omega +i\varepsilon }=P.V.\frac{i}{\omega }+\pi \delta \left(\omega \right).$$

Because of causality, the response function R(t)  must have the form R(t)=H(t)·M(t), where R(t)=M(t) for t>0. We have the freedom to choose the type of the memory function M(t) for t<0. The Fourier transform of the equation R(t)=H(t)·M(t) has the form

(45) $$\tilde{R}\left(\omega \right)=\frac{1}{2\pi }P.V.\overset{+\infty }{\underset{-\infty }{\int }}\frac{i}{\Omega -\omega }\tilde{M}\left(\Omega \right)d\Omega +\frac{1}{2}\tilde{M}\left(\omega \right).$$

Then we can use the freedom to choose the memory function M(t) for t<0. Considering the memory function M(t) as the even and odd functions, we can get the Kramers–Kronig relations [49].

Setting the function M(t) as even (M(−t)=M(t)), we get that $\tilde{M}\left(\omega \right)$ is a purely real quantity ${\tilde{M}}^{*}\left(\omega \right)=\tilde{M}\left(\omega \right)$. Then Equation (45) gives M˜(ω)=2·Re[R˜(ω)] that will be denoted as ${\tilde{M}}_{1}\left(\omega \right)$. Substitution of this expression to (45) gives

(46) $${\tilde{M}}_{2}\left(\omega \right)=-\frac{1}{\pi }P.V.\overset{+\infty }{\underset{-\infty }{\int }}\frac{1}{\Omega -\omega }{\tilde{M}}_{1}\left(\Omega \right)d\Omega .$$

Setting the function M(t) as odd (M(−t)=−M(t)), we get that $\tilde{M}\left(\omega \right)$ is a purely imaginary quantity ${\tilde{M}}^{*}\left(\omega \right)=\tilde{M}\left(\omega \right)$. Then Equation (45) gives i·M˜(ω)=−2·Im[R˜(ω)] that will be denoted as ${\tilde{M}}_{2}\left(\omega \right)$. Substitution of this expression to (45) gives

(47) $${\tilde{M}}_{1}\left(\omega \right)=\frac{1}{\pi }P.V.\overset{+\infty }{\underset{-\infty }{\int }}\frac{1}{\Omega -\omega }{\tilde{M}}_{2}\left(\Omega \right)d\Omega .$$

The memory function $\tilde{M}\left(\omega \right)$ is the complex function

(48) $$\tilde{M}\left(\omega \right)={\tilde{M}}_{1}\left(\omega \right)+i\cdot {\tilde{M}}_{2}\left(\omega \right),$$

where M˜1(ω)=Re[M˜(ω)]  and M˜2(ω)=Im[M˜(ω)] are real-valued functions.

In this section, we limited our consideration of the causality principle only to its effect on the property of the memory function for time and frequency domains. Note that the causality in economics is also discussed in [73,74,75,76,77].

## 6. Examples: Multipliers and Accelerators with Power-Law Memory

Let us give examples of multipliers and accelerators with power-law memory by using the fractional integrals and derivatives of non-integer orders [31,32,33]. For simplification, we will use the Liouville fractional integrals and derivatives. Note that we can use the Marchaud fractional derivative instead of the Liouville fractional derivative [31] (pp. 93–119). The Marchaud fractional derivative is more convenient than the Liouville fractional derivative, since they allow more freedom for the function X(t) at infinity [31] (p. 110). The multipliers and accelerators with memory, which are described by other types of fractional derivatives and integrals, are considered in [18,19,21,22].

### 6.1. Multipliers with Power-Law Memory

The economic multiplier with power-law memory can be described [18,19,21,22] by the fractional integral equation of the order α > 0 in the form

(49) $$Y\left(t\right)=m\left(\alpha \right)\cdot (I_{L;+}^{\alpha }X)\left(t\right),$$

where m(α) is the multiplier coefficient, and $I_{L;+}^{\alpha }$ is the left-sided Liouville integral of the order α > 0 with respect to time variable. This integral is defined in [60] (pp. 93–119), [62] (p. 87) by the equation

(50) $$\left(I_{L;+}^{\alpha }X\right)\left(t\right):=\frac{1}{\Gamma \left(\alpha \right)}\int_{-\infty }^{t}\frac{X\left(\tau \right)d\tau }{{\left(t-\tau \right)}^{1-\alpha }}=\frac{1}{\Gamma \left(\alpha \right)}\int_{-\infty }^{t}{\left(t-\tau \right)}^{\alpha -1}\cdot X\left(\tau \right)d\tau ,$$

where Γ(α) is the Gamma function. The Liouville integral (50) is a generalization of the standard integration [31,32,33]. In order to have the correct dimensions of economic quantities, we will use the dimensionless time variable t.

In the equations of multipliers with power-law memory, which are discussed above, the power-law fading is characterized by one parameter α≥0 only. In economic models we can take into account the presence of different types of memory fading, which characterize the different types of economic agents [18,19,21]. In the simplest multi-parametric case, the multiplier with fading memory can be considered as a sum of multipliers with one-parametric memory fading. For example, we can use the equation of the linear multiplier with N-parametric power-law memory [21,22] in the form:

(51) $$Y\left(t\right)=\overset{N}{\underset{k=1}{\sum }}m_{k}\left(\alpha \right)\cdot \left(I_{L;0+}^{\alpha_{k}}X\right)\left(t\right),$$

where $\alpha_{N}>\ldots >\alpha_{1}>0,$ and $m_{k}\left(\alpha \right)$ (k=1, 2, …N) are numerical coefficients. Using the memory with multi-parametric power-law fading, we can consider economic models that allow us to describe the memory effects in economy with different type of economic agents.

The Fourier transform (1) of the Liouville fractional integral (50) has the power-law form (see [31] (p. 137) and [32] (p. 90)) that is given by equation

(52) $$F\left\{I_{L;+}^{\alpha }X\left(t\right)\right\}\left(\omega \right)={\left(-i\omega \right)}^{-\alpha }\cdot F\left\{X\left(t\right)\right\}\left(\omega \right),$$

where we use a positive sign in front i·ω·t in the Fourier transform (1) and

(53) $${\left(-i\omega \right)}^{\alpha }={\left|\omega \right|}^{\alpha }\cdot \exp\left(-i\cdot \alpha \cdot \pi \cdot \frac{\mathrm{sgn}\left(\omega \right)}{2}\right).$$

For ω > 0, Equation (53) has the form

(54) $${\left(-i\omega \right)}^{\alpha }=\omega^{\alpha }\cdot \left(\cos\left(\frac{\pi \alpha }{2}\right)-i\cdot \sin\left(\frac{\pi \alpha }{2}\right)\right).$$

Using (52), the Fourier transform of Equation (49) takes the form

(55) $$\tilde{Y}\left(\omega \right)=m\left(\alpha \right)\cdot {\left(-i\omega \right)}^{-\alpha }\cdot \tilde{X}\left(\omega \right),$$

where $\tilde{Y}\left(\omega \right)=F\left\{Y\left(t\right)\right\}\left(\omega \right)$ and $\tilde{X}\left(\omega \right)=F\left\{X\left(t\right)\right\}\left(\omega \right)$. As a result, the memory function in the frequency domain has the form $\tilde{M}\left(\omega \right)=m\left(\alpha \right)\cdot {\left(-i\omega \right)}^{-\alpha }$.

Equation (55) allows us to state that the Fourier transform of the multiplier with memory has the power-law form exactly.

In the frequency domain, the linear multiplier with N-parametric power-law memory has the form

(56) $$\tilde{Y}\left(\omega \right)=\overset{N}{\underset{k=1}{\sum }}m_{k}\left(\alpha \right)\cdot {\left(-i\omega \right)}^{-\alpha_{k}}\cdot \tilde{X}\left(\omega \right),$$

where $\alpha_{N}>\ldots >\alpha_{1}>0,$ and $m_{k}\left(\alpha \right)$ (k=1, 2, …N) are numerical coefficients. As a result, we get

(57) $$\tilde{M}\left(\omega \right)=\frac{\tilde{Y}\left(\omega \right)}{\tilde{X}\left(\omega \right)}=\overset{N}{\underset{k=1}{\sum }}m_{k}\left(\alpha \right)\cdot {\left(-i\omega \right)}^{-\alpha_{k}},$$

where $-\alpha_{N}<\ldots <-\alpha_{1}<0$. Using that ${\tilde{M}}_{0}\left(\omega \right)=\tilde{M}\left(\omega \right)$, it is easy to get the limits

(58) $$\underset{\omega \to 0}{\lim}\frac{\ln\left|{\tilde{M}}_{0}\left(\omega \right)\right|}{\ln\left|\omega \right|}=-\alpha_{N},$$

(59) $$\underset{\omega \to \infty }{\lim}\frac{\ln\left|{\tilde{M}}_{\infty }\left(\omega \right)\right|}{\ln\left|\omega \right|}=-\alpha_{1}.$$

This means that $\alpha_{0}=-\alpha_{N}$ and $\alpha_{\infty }=-\alpha_{1}$, and we have a multiplier with multi-parametric PLT memory.

### 6.2. Accelerators with Power-Law Memory

The economic accelerator with one-parametric power-law memory [18,19,21,22] can be described by the fractional differential equation of the order α > 0 in the form

(60) $$Y\left(t\right)=a\left(\alpha \right)\cdot \left(D_{L;+}^{\alpha }X\right)\left(t\right),$$

where a(α) is the accelerator coefficient, and $D_{L;+}^{\alpha }$ is the left-sided Liouville fractional derivative integral of the order α>0 with respect to time variable, which is defined [31] (pp. 93–119), [32] (p. 87) by the equation

(61) $$\left(D_{L;+}^{\alpha }X\right)\left(t\right):=\frac{1}{\Gamma \left(n-\alpha \right)}\frac{d^{n}}{{\mathrm{dt}}^{n}}\int_{-\infty }^{t}\frac{X\left(\tau \right)d\tau }{{\left(t-\tau \right)}^{\alpha -n+1}}=\frac{1}{\Gamma \left(n-\alpha \right)}\frac{d^{n}}{{\mathrm{dt}}^{n}}\int_{-\infty }^{t}{\left(t-\tau \right)}^{n-\alpha -1}\cdot X\left(\tau \right)d\tau ,$$

where n = [α] + 1 and τ < t. Here function X(τ) must have the derivatives of integer orders up to the (n − 1) order, which are absolutely continuous functions on the interval (−∞,t].

Accelerator Equation (60) contains the standard equation of the accelerator and the multiplier, as special cases. For example, using the property $\left(D_{L;+}^{1}X\right)\left(t\right)=X^{\left(1\right)}\left(t\right)$ of the Liouville fractional derivative (see Equation (2.3.5) of [32] (p. 87)). Equation (60) with α = 1 gives equation $Y\left(t\right)=a\left(1\right)\cdot X^{\left(1\right)}\left(t\right)$ that describes the standard accelerator. Using the property $\left(D_{L:+}^{0}X\right)\left(t\right)=X\left(t\right)$ of the Liouville fractional derivative (see Equation (2.3.5) of [32] (p. 87)), Equation (60) with α = 0 can be written as Y(t) = a(0)·X(t), that is a standard equation of multiplier. As a result, the accelerator with memory (60) generalizes the standard economic concepts of the accelerator and the multiplier [21,22].

In the simplest linear accelerator with power-law memory, the power-law fading is characterized by one parameter α only. In general, different types of economic agents can be characterized by different types of memory fading. In this case, we can take into account N parameters of memory fading by using the equation

(62) $$Y\left(t\right)=\overset{N}{\underset{k=1}{\sum }}a_{k}\left(\alpha_{k}\right)\cdot \left(D_{L;+}^{\alpha_{k}}X\right)\left(t\right),$$

where $a_{k}\left(\alpha_{k}\right)$ are numerical coefficients and $\alpha_{N}>\ldots >\alpha_{1}>0$. The use of the accelerators with multi-parametric power-law memory allows us to describe the memory effects in economics with different types of economic agents.

It should be emphasized that the Fourier transform of the Liouville fractional integral and derivative has the power-law form (see [31] (p. 137) and [32] (p. 90). The Fourier transform of the Liouville fractional derivative (61) is represented by the expression

(63) $$F\left\{D_{L;+}^{\alpha }X\left(t\right)\right\}\left(\omega \right)={\left(-i\omega \right)}^{\alpha }\cdot F\left\{X\left(t\right)\right\}\left(\omega \right),$$

where

(64) $${\left(-i\omega \right)}^{\alpha }={\left|\omega \right|}^{\alpha }\cdot \exp\left(-i\cdot \alpha \cdot \pi \cdot \frac{\mathrm{sgn}\left(\omega \right)}{2}\right).$$

Using (63), we get the Fourier transform of the accelerator with memory (60) in the form

(65) $$\tilde{Y}\left(\omega \right)=a\left(\alpha \right)\cdot {\left(+i\omega \right)}^{\alpha }\cdot \tilde{X}\left(\omega \right).$$

where $\tilde{Y}\left(\omega \right)=F\left\{Y\left(t\right)\right\}\left(\omega \right)$ and $\tilde{X}\left(\omega \right)=F\left\{X\left(t\right)\right\}\left(\omega \right)$. Equation (65) allows us to state that the Fourier transform of the accelerator with memory has the power-law form exactly. As a result, the memory function in the frequency domain has the form $\tilde{M}\left(\omega \right)=a\left(\alpha \right)\cdot {\left(i\omega \right)}^{\alpha }$.

In the frequency domain, the accelerator with N-parameter memory has the form

(66) $$\tilde{Y}\left(\omega \right)=\overset{N}{\underset{k=1}{\sum }}a_{k}\left(\alpha \right)\cdot {\left(i\omega \right)}^{\alpha_{k}}\cdot \tilde{X}\left(\omega \right),$$

where $a_{k}\left(\alpha \right)$ (k=1, 2, …N) are numerical coefficients and $\alpha_{N}>\ldots >\alpha_{1}>0$. As a result, we get

(67) $$\tilde{M}\left(\omega \right)=\frac{\tilde{Y}\left(\omega \right)}{\tilde{X}\left(\omega \right)}=\overset{N}{\underset{k=1}{\sum }}a_{k}\left(\alpha \right)\cdot {\left(i\omega \right)}^{\alpha_{k}}.$$

Using that ${\tilde{M}}_{0}\left(\omega \right)=\tilde{M}\left(\omega \right)$ and $\alpha_{N}>\ldots >\alpha_{1}>0$, we get

(68) $$\underset{\omega \to 0}{\lim}\frac{\ln\left|{\tilde{M}}_{0}\left(\omega \right)\right|}{\ln\left|\omega \right|}=\alpha_{1},$$

(69) $$\underset{\omega \to \infty }{\lim}\frac{\ln\left|{\tilde{M}}_{\infty }\left(\omega \right)\right|}{\ln\left|\omega \right|}=\alpha_{N}.$$

This means that $\alpha_{0}=\alpha_{1}$ and $\alpha_{\infty }=\alpha_{N}$, where $\alpha_{\infty }>\alpha_{0}$. This proves that we have accelerator with multi-parametric PLT memory.

### 6.3. Generalized Accelerators with Power-Law Memory

We can consider the generalized accelerator with multi-parametric power-law memory that is defined by the equation

(70) $$\overset{m}{\underset{l=0}{\sum }}b_{l}\left(\beta \right)\cdot \left(D_{L;+}^{\beta_{l}}Y\right)\left(t\right)=\overset{n}{\underset{k=0}{\sum }}a_{k}\left(\alpha \right)\cdot \left(D_{L;+}^{\alpha_{k}}X\right)\left(t\right),$$

where $a_{k}\left(\alpha_{k}\right)$, $b_{l}\left(\beta_{l}\right)$ are numerical coefficients and $\alpha_{n}>\ldots >\alpha_{1}>\alpha_{0}=0$, $\beta_{m}>\ldots >\beta_{1}>\beta_{0}=0$. In the frequency domain, this generalized accelerator with memory has the form

(71) $$\overset{m}{\underset{l=0}{\sum }}b_{l}\left(\beta \right)\cdot {\left(i\omega \right)}^{\beta_{l}}\cdot \tilde{Y}\left(\omega \right)=\overset{n}{\underset{k=0}{\sum }}a_{k}\left(\alpha \right)\cdot {\left(i\omega \right)}^{\alpha_{k}}\cdot \tilde{X}\left(\omega \right).$$

As a result, we get

(72) $$\tilde{M}\left(\omega \right)=\frac{\tilde{Y}\left(\omega \right)}{\tilde{X}\left(\omega \right)}=\frac{\sum_{k=0}^{n}a_{k}\left(\alpha \right)\cdot {\left(i\omega \right)}^{\alpha_{k}}}{\sum_{l=0}^{m}b_{l}\left(\beta \right)\cdot {\left(i\omega \right)}^{\beta_{l}}}.$$

Using ${\tilde{M}}_{0}\left(\omega \right)=\tilde{M}\left(\omega \right)-\tilde{M}\left(0\right)=\tilde{M}\left(\omega \right)-a_{0}\left(\alpha \right)/b_{0}\left(\beta \right)$ for the case $a_{0}\left(\alpha \right)\neq 0$ and $b_{0}\left(\beta \right)\neq 0$, we get

(73) $$\underset{\omega \to 0}{\lim}\frac{\ln\left|{\tilde{M}}_{0}\left(\omega \right)\right|}{\ln\left|\omega \right|}=\alpha_{1}-\beta_{1}.$$

Equation (73) means that $\alpha_{0}=\alpha_{1}-\beta_{1}$. This proves that we have accelerator with multi-parametric PLT memory if the parameter $\alpha_{0}=\alpha_{1}-\beta_{1}$ takes non-integer value.

The limit ω→∞ gives

(74) $$\underset{\omega \to \infty }{\lim}\frac{\ln\left|{\tilde{M}}_{\infty }\left(\omega \right)\right|}{\ln\left|\omega \right|}=\alpha_{n}-\beta_{m}.$$

Equation (74) means that $\alpha_{\infty }=\alpha_{n}-\beta_{m}$. In this case, we have the multiplier with multi-parametric PLT memory for the case $\alpha_{n}-\beta_{m}<0$ and the accelerator with PLT memory for the case $\alpha_{n}-\beta_{m}>0$ if the parameter $\alpha_{\infty }=\alpha_{n}-\beta_{m}$ takes non-integer values.

## 7. Examples: Generalized Memory Functions of Power-Law Type

Using the econophysics approach, we consider the memory function, which cannot be considered as power-law memory exactly. This function describes memory of the power-law type. This example of the memory function we take from physics, more precisely from electrodynamics, but it can be used to describe economic processes with memory.

In electrodynamics of dielectric media, the analog of exogenous variable X(t) is the electric field $\vec{E}\left(t,\vec{r}\right),$ the analog of endogenous variable Y(t) is the polarization density $\vec{P}\left(t,\vec{r}\right),$ and the analog of the response (memory) function M(t) is the dielectric susceptibility χ(t). The dielectric susceptibility of most materials follows a fractional power-law frequency dependence that is called the universal relaxation law or ‘‘universal’’ response [35,36,37,38,39,40,41] (see also [42,43,44,45,46]).

The power-law type of behavior is confirmed in different measurements realized by Jonscher [35,36,37,38] for a wide class of media. Jonscher formulated the “universal” response phenomenon, when the branches of dielectric spectra (expressed in the form of complex susceptibility) are described by the expressions in the form:

(75) $$\tilde{M}\left(\omega \right)-\tilde{M}\left(0\right)\sim -{\left(i\cdot \omega \right)}^{\beta },\mathrm{for}\;\omega \to 0\;\left(\omega \ll \omega_{p}\right),\;\mathrm{where}\;0<\beta =m<1,$$

(76) $$\tilde{M}\left(\omega \right)\sim {\left(i\cdot \omega \right)}^{-\alpha }\;\mathrm{for}\;\omega \to \infty \;\left(\omega \gg \omega_{p}\right),\;\mathrm{where}\;0<\alpha =1-n<1.$$

The parameter β=m for $\omega \to 0\;\left(\omega \ll \omega_{p}\right),$ and the parameter α=1−n for $\omega \to \infty \;\left(\omega \gg \omega_{p}\right),$ where α and β fall in the range (0;1). The law is based on the experimental observation of power laws at frequencies far from the peak frequency of the imaginary part ${\tilde{M}}_{2}\left(\omega \right)$ of the frequency dependent response (memory) function $\tilde{M}\left(\omega \right)$, the susceptibility. A general (“universal”) rule of frequency dependence of the response (memory) function is described by fractional power laws. 

The Jonscher equation [35] has two shape parameters and is formulated for the imaginary part ${\tilde{M}}_{2}\left(\omega \right)$ in the form

(77) $${\tilde{M}}_{2}\left(\omega \right)=\frac{1}{{\left(\omega /\omega_{p}\right)}^{\alpha }+{\left(\omega /\omega_{p}\right)}^{-\beta }},$$

where $\omega_{p}$ is the loss peak frequency. The behavior of Function (77) is described by (75) and (76).

A purely empirical analytical expression, which is convenient for representing the generalized loss peaks, was proposed by Havriliak and Negami [42] in 1967 and is given by

(78) $$\tilde{M}\left(\omega \right)=\frac{1}{{\left(1+{\left(i\cdot \omega /\omega_{p}\right)}^{\alpha }\right)}^{\gamma }}$$

where α=m and γ=(1−n)/m, where $\omega_{p}$ is the loss peak frequency. The Havriliak–Negami equation [71] also has two shape parameters but, in contrast to the Jonscher equation, it is formulated directly for the complex function $\tilde{M}\left(\omega \right)$. The widely used Havriliak–Negami equation [71] with two shape parameters describes most observed responses. For γ=1 the Havriliak–Negami equation reduces to the Cole–Cole equation, for α=1 to the Cole–Davidson equation.

The Havriliak–Negami Function (78) has the correct asymptotic behavior for ω→0 and ω→∞. This function is actively used to approximate the experimental data in electrodynamics. A comprehensive presentation of this empirical function and the determination of the parameters is given in [72].

As a result, using (75) and (76), we get

(79) $$\underset{\omega \to 0}{\lim}\frac{\ln\left|{\tilde{M}}_{0}\left(\omega \right)\right|}{\ln\left|\omega \right|}=\beta ,$$

(80) $$\underset{\omega \to \infty }{\lim}\frac{\ln\left|{\tilde{M}}_{\infty }\left(\omega \right)\right|}{\ln\left|\omega \right|}=\alpha .$$

This means that $\alpha_{0}=\beta$ and $\alpha_{\infty }=\alpha$. This proves that we have accelerator with multi-parametric PLT memory.

In the time domain, the Havriliak–Negami function (the corresponding time-domain relaxation function) can be represented through the H-function [42] or three parameters Mittag–Leffler function [75] (p. 223) by the equation

(81) $$M\left(t\right)=1-{\left(\omega_{p}\cdot t\right)}^{\alpha \cdot \gamma }\cdot E_{\alpha ,\alpha \cdot \gamma +1}^{\gamma }\left[-{\left(\omega_{p}\cdot t\right)}^{\alpha }\right],$$

where $E_{\alpha ,\beta }^{\gamma }\left[z\right]$ is the three parameters Mittag–Leffler function (for example, see [32] (p. 45), [79] (pp. 97–126)) that is defined by expression

(82) $$E_{\alpha ,\beta }^{\gamma }\left[z\right]:=\sum_{k=0}^{\infty }\frac{\Gamma \left(\gamma +k\right)}{\Gamma \left(\gamma \right)\cdot \Gamma \left(k+1\right)}\cdot \frac{z^{k}}{\Gamma \left(\alpha \cdot k+\beta \right)},$$

where α > 0, and β, γ are arbitrary real or complex numbers. The three–parametric Mittag-Leffler function can be considered as a special case of the H-function by the equation

(83) $$E_{\alpha ,\beta }^{\gamma }\left[z\right]=\frac{1}{\Gamma \left(\gamma \right)}\cdot H_{1\;2}^{1\;1}\left[-z|\begin{array}{c} \left(1-\gamma ,\;1\right)\\ \left(0,1\right)\left(1-\beta ,\alpha \right) \end{array}\right].$$

For details see [32], (p. 67) and [79] (p. 105).

The memory Function (83) can be used to describe economic processes with memory that is characterized by the frequency behavior in the form (75) and (76).

## 8. Examples: ARFIMA Models and the Grunwald–Letnikov Fractional Difference

In economics, the memory was first related to fractional differencing and integrating by C.W.J. Granger, R. Joyeux [14], and Hosking, J.R.M. [80] by using the discrete time approach for models with t∈ℤ. Granger and Joyeux, and Hosking independently propose the so-called autoregressive fractional integrated moving average models (ARFIMA models). The difference operator used in this model is

(84) $$\Delta^{d}:={\left(1-L\right)}^{d},$$

where L is the lag operator LX(t)=X(t−1), and d is the order of the fractional differencing (integrating) [14,80], which need not be an integer. In papers [8,9] we noted that the operator (84) is the difference of fractional (integer or non-integer) order, which is called the Grunwald–Letnikov fractional difference of the order d with the unit step T=1 (for example, see Section 20 of [31] (pp. 371–387) and [32] (pp. 121–123), [33] (pp. 43–62)). The Grunwald–Letnikov fractional difference $\Delta_{T}^{\alpha }$ of order α with the step T is defined by the equation

(85) $$\Delta_{T}^{\alpha }X\left(t\right):={\left(1-L_{T}\right)}^{\alpha }X\left(t\right)=\overset{\infty }{\underset{m=0}{\sum }}{\left(-1\right)}^{m}\cdot \left(\begin{array}{c} d\\ m \end{array}\right)\cdot X\left(t-m\cdot T\right),$$

where $L_{T}X\left(t\right)=X\left(t-T\right)$ is the fixed-time delay operator and the time-constant T is any given positive value. The series (85) convergences absolutely and uniformly for each α>0 and for every bounded function X(t). As a result, the description of the ARFIMA models is based on the operators that are the well-known Grunwald–Letnikov fractional differences (85) of the order α=d. Note that the difference (85) can be used for continuous time models, where t∈ℝ.

The expression ${\left(1-L\right)}^{d}$ can be defined by the series expansion, [31] (p. 371), in the form

(86) $${\left(1-L_{T}\right)}^{d}:=\overset{\infty }{\underset{m=0}{\sum }}{\left(-1\right)}^{m}\cdot \left(\begin{array}{c} d\\ m \end{array}\right)\cdot L_{T}^{m},$$

where $\left(\begin{array}{c} d\\ m \end{array}\right)$ are the generalized binomial coefficients (see Equation (1.50) of [31] (p. 14)), that are defined by the equation

(87) $$\left(\begin{array}{c} d\\ m \end{array}\right):=\frac{\Gamma \left(d+1\right)}{\Gamma \left(d-m+1\right)\cdot \Gamma \left(m+1\right)},$$

where Γ(z) is the Euler gamma function.

Using Equation (1.48) of [31] (p. 14), the binomial coefficients $\left(\begin{array}{c} d\\ m \end{array}\right)$ can be written in the form

(88) $$\left(\begin{array}{c} d\\ m \end{array}\right):=\frac{{\left(-1\right)}^{m-1}\cdot d\cdot \Gamma \left(m-d\right)}{\Gamma \left(1-d\right)\cdot \Gamma \left(m+1\right)}.$$

Using Expression (88), Equation (86) can be represented in the following form

(89) $${\left(1-L_{T}\right)}^{d}=\overset{\infty }{\underset{m=0}{\sum }}\frac{\Gamma \left(m-d\right)}{\Gamma \left(-d\right)\cdot \Gamma \left(m+1\right)}\cdot L_{T}^{m},$$

which is usually used in the econometric papers on long memory and time series. 

As a result, Equation (86) is the fractional difference equation. Equation (86) can be generalized for the continuous time case by using the fractional difference equation

(90) $$Y\left(t\right)=a\left(\alpha \right)\cdot \Delta_{T}^{\alpha }X\left(t\right).$$

It should be noted that the Grunwald–Letnikov fractional difference (86) may converge for α<0, if Y(t) has a “good” decrease at infinity [31] (p. 372). For example, we can use the functions X(t) such that $\left|X\left(t\right)\right|\leq c\cdot {\left(1+\left|t\right|\right)}^{-\mu }$, where μ > |α|. This allows us to use (10) as a discrete fractional integration in the non-periodic case. 

In the non-periodic case the Fourier transform F of $\Delta_{T}^{\alpha }y\left(t\right)$ is given, (see Equation (20.3) of [31] (p. 373), by the equation

(91) $$F\left\{\Delta_{T}^{\alpha }X\left(t\right)\right\}\left(\omega \right)={\left(1-\exp\left(i\omega T\right)\right)}^{\alpha }F\left\{X\left(t\right)\right\}\left(\omega \right),$$

provided that X(t) is in the function space $L_{1}\left(R\right).$

Note that the Grunwald–Letnikov fractional difference $\Delta_{T}^{\alpha }$ of the order α cannot be considered as an exact fractional difference [8,9] (see also [23,61,62]), since the Fourier transforms of the Grunwald–Letnikov fractional differences are not the power law, i.e., $F\left\{\Delta_{T}^{\alpha }X\left(t\right)\right\}\left(\omega \right)\neq {\left(i\omega T\right)}^{\alpha }F\left\{X\left(t\right)\right\}\left(\omega \right)$.

In the simplest linear accelerator with the PLT memory, the power-law fading is characterized by the one parameter α>0 only and it has the form

(92) $$Y\left(t\right)=a\left(\alpha \right)\cdot \Delta_{T}^{\alpha }X\left(t\right),$$

where a(α) is numerical coefficient. In the frequency domain, the accelerator with memory has the form

(93) $$\tilde{Y}\left(\omega \right)=a\left(\alpha \right)\cdot {\left(1-\exp\left(i\omega T\right)\right)}^{\alpha }\cdot \tilde{X}\left(\omega \right),$$

where and $a_{k}\left(\alpha \right)$ (k=1, 2, …N) are numerical coefficients and $\alpha_{N}>\ldots >\alpha_{1}>0$. As a result, we get

(94) $$\tilde{M}\left(\omega \right)=\frac{\tilde{Y}\left(\omega \right)}{\tilde{X}\left(\omega \right)}=a\left(\alpha \right)\cdot {\left(1-\exp\left(i\omega T\right)\right)}^{\alpha }.$$

Using that ${\tilde{M}}_{0}\left(\omega \right)=\tilde{M}\left(\omega \right)$ and α>0, we get

(95) $$\underset{\omega \to 0}{\lim}\frac{\ln\left|{\tilde{M}}_{0}\left(\omega \right)\right|}{\ln\left|\omega \right|}=\alpha ,$$

(96) $$\underset{\omega \to \infty }{\lim}\frac{\ln\left|{\tilde{M}}_{\infty }\left(\omega \right)\right|}{\ln\left|\omega \right|}=0.$$

Equations (95) and (96) mean that $\alpha_{0}=\alpha$ and $\alpha_{\infty }=0$. Note that this is an example, for which $\alpha_{\infty }<\alpha_{0}$. We prove that we have accelerator with PLT memory.

## 9. Conclusions

In this paper, the criteria for the existence of memory of power-law type in economic processes are proposed. Implementation of the proposed criteria for dependencies of endogenous and exogenous variables in an economic process allows use of fractional calculus for the construction of dynamic models of the economic process. The proposed criteria can be important for specifying the class of differential and integral operators that can be used to describe economic processes with memory of power-law type. As a result, the suggested approach can be used for constructing models of real financial and economic processes, [81,82,83,84,85,86,87,88,89] and [19,25,26,27,28,29,30,89,90,91,92,93,94,95], in which memory effects are manifested. Note that phenomenological economic process are described by computer simulation of the economies of Spain, Portugal, France, Italy are presented by I. Tejado, D. Valerio, E. Perez, and N. Valerio in the works [89,90,91,92].

An important direction in the development of economic models with memory is the construction of models, in which the economic agents are represented as a self-organizing set of interacting agents with memory. Note that the power-law long-range correlations can be connected with critical phenomena and self-organization processes in natural sciences [96,97].

In papers [19,25,94], we prove that the rates of technological growth with PLT memory (which is a generalization of the Harrod’s “warranted” rate of growth [98], (p. 67)) do not coincide with the growth rates λ of standard models without memory. The rate of technological growth with memory is equal to the value $\lambda_{\mathrm{eff}}\left(\alpha \right):=\lambda^{1/\alpha }$. For the parameter α=1 we have the standard rate of growth without memory $\lambda_{\mathrm{eff}}\left(1\right)=\lambda$. We also prove [19,25,95] that the account of PLT memory can significantly change the technological growth rates in the macroeconomic model. For example, the memory effect can give a growth instead of decreasing and decline. The effects of PLT memory with α >1 can lead to economic growth instead of a recession economy. The PLT memory can also lead to significant change in growth rate. For example, for α=0.1 and λ = 2, the rate of technological growth with memory (α=0.1) is equal to $\lambda_{\mathrm{eff}}\left(0.1\right)=1024$ instead of $\lambda_{\mathrm{eff}}\left(1\right)=\lambda \;=\;2\;$for the standard model without memory (α=1), that is, 512 times more. This indicates that taking into account the PLT memory in the macroeconomic model can change the rate of technological growth by several orders of magnitude. Using the principle of domination change, which is proposed in [23] for intersectoral models, we can state that the inequality $\lambda_{1}<\lambda_{2}$, of the standard two sectoral model can leads to the inequality $\lambda_{1,\mathrm{eff}}\left(\alpha_{1}\right)>\lambda_{2,\mathrm{eff}}\left(\alpha_{2}\right)$ of the model with power-law sectoral memory. For example, $\lambda_{1}=2$ and $\lambda_{2}=3$ can lead to $\lambda_{1,\mathrm{eff}}\left(\alpha_{1}\right)=1024$ and $\lambda_{2,\mathrm{eff}}\left(\alpha_{2}\right)=\sqrt[{3}]{9}\approx 2.08$ for the memory parameter $\alpha_{1}=0.1$, $\alpha_{2}=1.5$. This fact allows us to use adiabatic approximation to intersectoral model with memory [25,94] and to interpret the memory fading parameter as an order parameter described in [99], (pp. 191–200). This fact can be used to construct economic models with self–organizing and memory as a union of the proposed approach to economic processes with memory and the synergetic economics [100,101].

In addition to applications of the proposed criteria to identify the presence of the PLT memory in processes, for which dependent variables are known, these criteria can be used to check whether linear integro-differential operators are fractional derivatives and integrals of non-integer orders. It is known that the wide class of PLT memory function allows the use of fractional calculus to describe processes with memory. In recent years some different type of new integral and differential operators, which do not relate to the fractional calculus, are suggested. Some of these operators cannot be used to describe a memory since it cannot be considered as non-local operators [34]. The memory can be interpreted as a nonlocality in time, and therefore we can state “No memory. No fractional derivatives and integrals”. Because of this, the question arises about the possibility of building a type of functions, which is broader than power-law functions, to describe a memory, and apply the fractional calculus or its generalization. We call these functions as functions of power-law type. Therefore the proposed criteria can be applied to develop the fractional calculus. The proposed criteria can be used to identify the linear integro-differential operators that can be considered as fractional derivatives and integrals of non-integer order. It allows to propose new types of fractional derivatives and integrals of non-integer orders, and this may lead to an expansion of the area of generalized fractional calculus [56,57] and its possible application.

## Figures and Tables

**Table 1:** Tables of interpretation of different signs and non-integer values of parameters $\alpha_{0}$ and $\alpha_{\infty }$, when there are only parameters $\alpha_{\infty }$, $\alpha_{0}$ and there are no other parameters ($\alpha_{0,k}$, $\alpha_{\infty ,k}$), k≥1, k∈ℤ.

Sings	Values	Phenomenon (Equation)	Memory
$\alpha_{\infty }>0;\alpha_{0}>0$	non-integer	accelerator with PLT memory	multi-parametric
$\alpha_{\infty }=\alpha_{0}>0$	non-integer	accelerator with PLT memory	one-parametric
$\alpha_{\infty }=0;\alpha_{0}>0$ or $\alpha_{\infty }>0;\alpha_{0}=0$	non-integer and integer	accelerator with PLT memory and without memory	one-parametric
$\alpha_{\infty }\in Z;\alpha_{0}>0$ or $\alpha_{\infty }>0;\alpha_{0}\in \mathbb{Z}$	non-integer and integer	accelerator with PLT memory and without memory	one-parametric
$\alpha_{\infty }=\alpha_{0}=1$	integer	accelerator without PLT memory	no memory
$\alpha_{\infty }=\alpha_{0}=0$	zero	multiplier without PLT memory	no memory
$\alpha_{\infty }>0>\alpha_{0}\;$ $\alpha_{0}>0>\alpha_{\infty }$	non-integer	accelerator-multiplier with PLT memory	multi-parametric
$\alpha_{0}<0\;;\alpha_{\infty }<0$	non-integer	multiplier with PLT memory	multi-parametric
$\alpha_{\infty }=\alpha_{0}<0$	non-integer	multiplier with PLT memory	one-parametric
